# Widespread Metastasis to the Stomach 10 Years After Primary Breast Cancer: A case report and review of the literature

**DOI:** 10.1097/MD.0000000000022527

**Published:** 2020-11-25

**Authors:** Ji-Xin Fu, Ya-Nan Zou, Long-Li, Xin-Jian Wang

**Affiliations:** a Department of Gastrointestinal Surgery; b Department of Anesthesiology; c Department of Gastrointestinal Surgery; d Department of Gastrointestinal Surgery, Weihai Central Hospital, Weihai, Shandong, China.

**Keywords:** breast cancer, chemokines, gastric metastases, GCDFP-15

## Abstract

**Rationale:**

Breast cancer is a common malignant tumor. The most common metastatic sites of breast cancer are the bone, brain, liver and lung, and gastrointestinal metastases are rare. Considering that the median time interval from the initial breast cancer diagnosis to stomach metastasis is 77.5 months, gastrointestinal metastases are rarely observed 10 years after primary breast cancer.

**Patient concerns:**

Here, we present a 63-year-old female with unusual endoscopy results that revealed scattered polyps and mucosal infiltration throughout the stomach, which were later confirmed to be metastatic lobular carcinoma of the breast that had been surgically removed 10 years earlier.

**Diagnosis:**

The patient was diagnosed with gastric metastases of breast cancer by immunohistochemistry.

**Interventions:**

The patient underwent endocrine therapy with palbociclib and tamoxifen.

**Outcomes:**

After 1 year of endocrine therapy, the symptoms of upper abdominal discomfort and fatigue were relieved and a new gastroscopy revealed there had been no significant progression of the gastric metastasis. According to the Response Evaluation Criteria in Solid Tumors (RECIST), the patient reached a state of stable disease.

**Lessons:**

Gastric metastases of breast cancer are rare in the clinical setting. However, considering the possibility of gastric metastases from breast cancer and performing an upper endoscopy are crucial for patients who present with any subtle gastric symptoms and have a past medical history of breast cancer, even if the breast cancer occurred more than 10 years ago.

## 1 Introduction

Breast cancer is a common malignant tumor that seriously threatens human health and quality of life. According to the latest statistics, breast cancer had the highest incidence rate and was the fifth leading cause of cancer-associated mortality worldwide in 2018, and the incidence of breast cancer is continuously increasing.^[1]^ It is well known that the most common distant metastatic sites of breast cancer are the bone, brain, liver, and lung. Gastric metastases from a breast malignancy are rare and are identified in only 0.3% of all gastrectomy specimens.^[2]^ The median time interval from a breast cancer diagnosis to gastric metastasis is 77.5 months, and an interval >5 years may be long enough to consider a gastric malignancy as a primary cancer,^[3]^ thus, our presentation is highly unusual. In this article, we present an uncommon case of a patient who presented with upper abdominal discomfort and fatigue and was confirmed to have gastric metastases from primary breast cancer that was surgically removed 10 years earlier.

## 2 Case report

**Figure 1:**
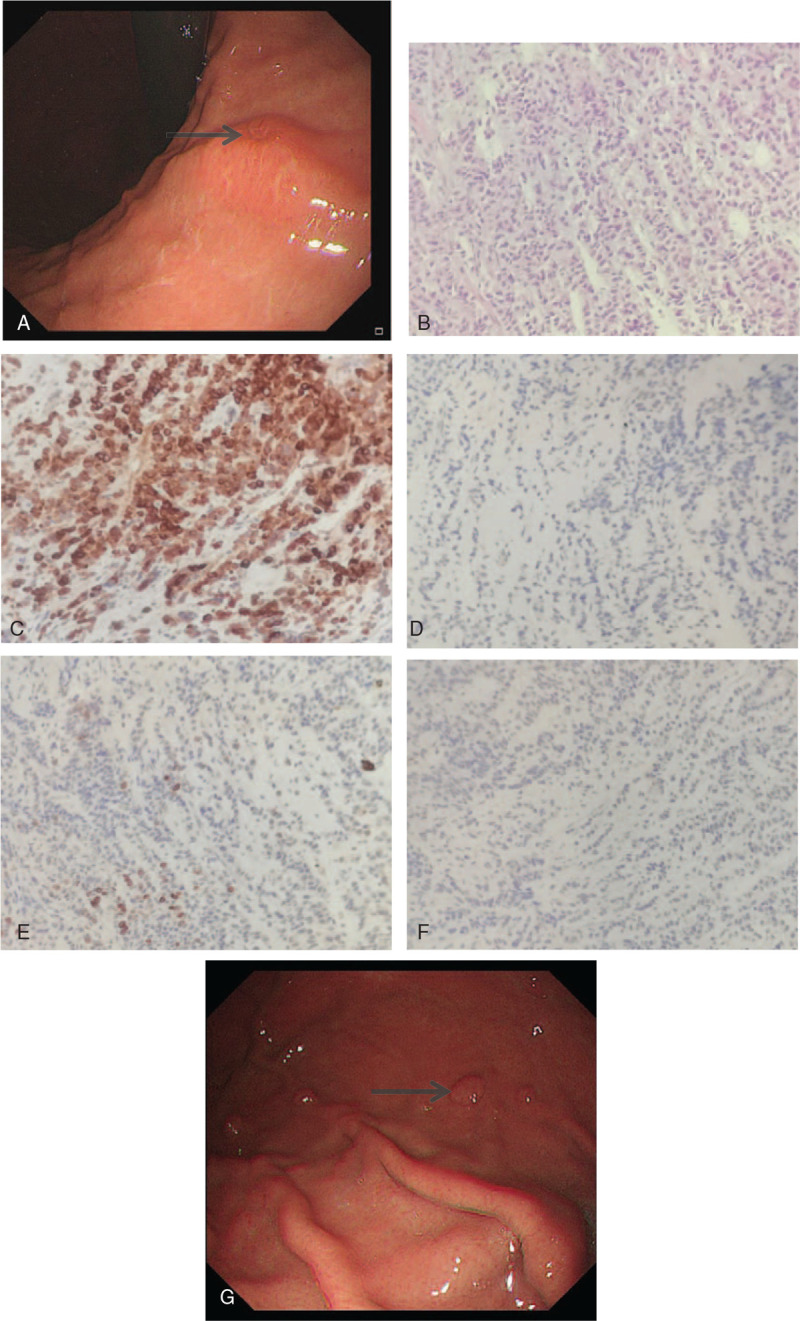
A. Endoscopy revealed scattered polyps and mucosal infiltration. B. (gastric body) Poorly differentiated adenocarcinoma (H&E stain, 200× magnification). C. Positive nuclear staining for GCDFP-15 (GCDFP-15 immunohistochemical stain, 200× magnification). D. Negative for CDX2 (CDX2 immunohistochemical stain, 200× magnification). E. Negative for CK20 (CK20 immunohistochemical stain, 200× magnification). F. Positive for CK7 (CK7 immunohistochemical stain, 200× magnification). G. A new gastroscopy revealed there was no significant progress with gastric metastasis.

A 63-year-old woman with a history of left breast cancer complained of upper abdominal discomfort and fatigue. Ten years prior, the patient had undergone a modified radical mastectomy for left breast cancer. The histopathological examination revealed lobular infiltrating carcinoma (S:1.5 × 1.0 cm); no cancer cells were found in the deep thoracic regions, papilla, or 15 lymph nodes of the axilla, and the TNM classification was T1cN0M0 stage IA. Immunohistochemical staining was highly positive for estrogen receptors (ER) and progestogen receptors (PRs), while the human epidermal growth factor receptor 2 (HER2) and p53 stains were negative. The patient received adjuvant chemotherapy with 75 mg/m^2^ epirubicin and 600 mg/m^2^ cyclophosphamide every 21 days for 4 cycles, followed by 6 years of 2.5 mg/d letrozole as endocrine therapy. She underwent regular follow-up and remained disease-free until 2018. One year prior, because of a pain in her waist, she was admitted to our hospital and she was diagnosed with bone metastasis after a whole-body bone scan and bone marrow biopsy. The patient underwent radiotherapy (DT40Gy/20f, 5f/W) and endocrine therapy with fulvestrant (0.5 g/d). Upon physical examination, she had no palpable masses in the abdomen and had no palpable nodules in her right breast. Notably, levels of carcinoembryonic antigen were elevated by 9.05 μg/L. Upper endoscopy revealed scattered polyps and mucosal infiltration, involving the gastric antrum, body, and cardia (Fig. 1A). Histopathological examination showed (gastric body) poorly differentiated adenocarcinoma, and immunohistochemistry staining with intense positivity for ER, gross cystic disease fluid protein 15 (GCDFP-15) and cytokeratin 7 (CK7), as well as negative staining of cytokeratin 20 (CK20), caudal-related homeobox 2 (CDX2), and mammaglobin (Fig. 1B–F). A computer tomography (CT) scan of the thorax and abdomen did not show any other distant sites of metastases. In view of widespread metastasis of the stomach, the patient received palbociclib (125 mg/d) and tamoxifen (20 mg/d) as endocrine therapy. After 1 year of endocrine therapy, the discomfort in the upper abdomen and fatigue were relieved, and there was no further complaint of back pain. A new gastroscopy revealed there to be no significant progress with gastric metastasis (Fig. 1G). According to the Response Evaluation Criteria in Solid Tumors (RECIST), the patient reached a state of stable disease.

The study was reviewed and approved by Ethical Committee of Weihai Central Hospital. Written informed consent was obtained from the patient for publication of this case report and any accompanying images.

## 3 Discussion

It is well known that the most common metastatic sites of breast cancer are the bone, brain, liver, and lung. Due to the very rare occurrence of gastric metastases, the suspicion of metastases in the stomach is rarely taken into account in our clinical setting.^[4]^ Unusual gastric metastases have been reported from cutaneous melanomas, and carcinomas of the breast, lung, kidney, ovary, adrenal glands, salivary glands, thyroid, etc.^[4–8]^ It was estimated that breast cancer causes the most gastric metastases (28%), followed by lung cancer (24%), esophageal cancer (19%), renal cell carcinoma (8%), malignant melanoma (7%), and head and neck carcinomas (1%–6%).^[6,8]^ Although gastric metastases from breast malignancies are the most frequent, these metastases are identified in only 0.3% of all gastrectomy specimens.^[2]^ Lobular infiltrating breast cancer is also known to specifically give rise to gastric metastasis.^[9]^ Indeed, gastric metastasis is often a part of a disseminated disease, and other metastatic localizations can be found simultaneously or continuously. Mubarak et al^[10]^ reported 65% of bone metastases, 57% of peritoneal carcinomatosis and 5% of hepatic localizations are also associated with gastric metastases. In our case, metastasis to the bone was found 1 year before the gastric metastases.

The PubMed (https://www.ncbi.nlm.nih.gov/pubmed) databases were investigated between 2015 and 2020 to analyze the clinicopathological features and outcomes of patients with gastrointestinal metastases of breast carcinoma. The reference lists were screened to identify additional relevant studies, and a standardized form was used for data extraction. Finally, there were approximately 32 cases of gastrointestinal metastases of breast carcer.^[11–42]^ The patient's information was summarized in Table 1 and the clinicopathological features were analyzed in Table 2. The mean age of the patients with gastrointestinal metastases was 62.75 years (range: 39–84). The sex distribution almost was female, except 2 males (male:female = 1:15). On the aspect of primary breast cancer, the most type of breast cancer was invasive lobular carcinoma, accounting for 64.29%, which was consistent with the results of Jmour et al,^[9]^ while the invasive ductal carcinoma account for 32.14%, besides, there was a rare case of ductal carcinoma in situ. The most common clinical stage were stage II and stage III, accounting for 35% and 35%, respectively. ER receptor and PR receptor expression were described in 25 cases and 20 cases, while HER2 expression was rare in primary breast cancer, which was only in 3 cases. Endocrine therapy was the main adjuvant therapy for patients after surgery, which account for 75.86%. On the aspect of gastrointestinal metastases, the main metastatic location were stomach and colon, accounting for 37.5% and 28.13%, respectively. The median time from the diagnosis of the primary cancer to the onset of gastrointestinal metastases was 9.62 years. The most common positive expression of gastrointestinal metastases were also ER receptor and PR receptor, which comprise 86.67% and 46.67% of cases, respectively. The median time of follow-up was 25.53 months after the diagnosis.

**Table 1:** Gastrointestinal metastases of breast carcinoma reported in the literature between 2015 and 2020.

			Characteristics of breast cancer					Characteristics of gastrointestinal metastasis					
Author, year	Sex	Age	Type	Stage	Receptor (E/P/H)	Treatment	AT after operation	Location	Interval time^∗^	Symptom	Receptor (E/P/H)	Treatment	Time of follow-up
Mohamad Sharbatji 2019^[11]^	F	72	ILC	ND	+/+/−	O	C/E	Small bowel	10 Y	Bowel obstruction	−/−/−	O	Lost
Numan 2019^[12]^	F	44	ILC	IV	+/+/−	O	E	Small bowel, appendix	3 Y	Small bowel obstruction and appendicitis	ND	O	Lost
Güler 2018^[13]^	F	42	IDC	IV	ND	C	No	Stomach	1 M	Gastric perforation	+/+/−	O	ND
Mroz 2015^[14]^	F	80	ILC	IIA	+/+/−	O	E	Right colon	9 Y	Rectal bleeding	+/+/−	O	4 Y
Wilson 2015^[15]^	F	62	ILC	ND	ND	O	C/R	Esophagus	11 Y	Dysphagia	+/−/−	O	5 Y
Yim 2017^[16]^	F	65	ILC	ND	ND	O	C/R	Stomach	4 Y	Indigestion and epigastric discomfort	−/−/+	E	4 M
Kim 2015^[17]^	F	57	ILC	IV	+/−/−	ND	ND	Stomach	ND	Dyspepsia, dysphagia	+/−/−	ND	ND
Falco 2018^[18]^	F	67	ILC	IIIC	+/+/−	O	C	Right colon	14 Y	No	+/+/−	O	7 M
Jones 2018^[19]^	M	55	ND	ND	+/+/−	O	E	Colon	4 Y	Nausea, vomiting and abdominal pain	+/+/−	O	4 M
Katz 2018^[20]^	F	68	IDC	ND	+/+/+	O	C/E	Colon	22 Y	Abdominal pain and swelling	+/−/−	O	ND
Ricciuti 2016^[21]^	M	65	IDC	ND	+/−/+	O	E	Stomach	12 Y	Hematemesis, epigastric pain	−/−/−	O	4 M
Kumar 2018^[22]^	F	59	ND	IIA	−/−/−	O	C	Esophagus	15 Y	Hoarseness of voice, dysphagia	+/+/−	C/R	1 Y
Miyake 2019^[23]^	F	77	IDC	IIIA	+/+/−	O	E	Esophagus	21 Y	Dysphagia	+/−/−	C/R	16 M
Gangireddy 2019^[24]^	F	72	ILC	ND	ND	O	C/R/E	Small bowel	24 Y	Nausea, vomiting, abdominal pain	+/+/−	O	ND
Tang 2019^[25]^	F	67	IDC	IIb	+/+/−	O	C/R	Stomach	2 Y	Stomach and lower back pain	−/−/−	C	ND
Villa Guzmán 2017^[26]^	F	58	ILC	IIIA	+/+/−	O	C/R/E	Stomach, colon	20 Y	Nausea and epigastric pain	+/−/−	C/R/E	Die after 5 Y
Waseda 2015^[27]^	F	57	ILC	ND	ND	O	E/C	Stomach	5 Y	Epigastric discomfort	+/+/−	C	ND
Kim DH 2018^[28]^	F	39	ILC	IIIA	+/+/−	O	C	Stomach	2 M	Epigastric discomfort	+/+/−	C	41 M
Asmar 2018^[29]^	F	84	ND	ND	+/+/−	O	C/R/E	Stomach	20 Y	Dyspepsia and gastric fullness	+/−/−	E	ND
Wong 2016^[30]^	F	72	ILC	IIA	+/+/−	O	R/E	Stomach	4 Y	Gastric perforation	+/−/−	O	3 M
Liu 2018^[31]^	F	56	IDC	ND	+/+/−	O	C/R/E	Small bowel	16 Y	Abdominal pain, nausea and vomiting	+/−/−	O	2 Y
Cherian 2017^[32]^	F	79	IDC	IIIA	+/−/−	O	R/E	Rectum	10 Y	Abdominal, lower back and anal pain	+/−/−	E	Die after 2 M
Amberger 2018^[33]^	F	50	ILC	II	+/+/−	O	No	Colon	11M	Nausea, vomiting, abdominal pain	+/+/−	O	Lost
Asfari 2017^[34]^	F	56	ND	ND	+/−/−	O	C	Esophagus	16 Y	Dysphagia	+/−/−	E	4 M
Lau 2017^[35]^	F	61	ILC	IA	ND	O	E	Rectum	11 Y	Change in bowel habits	+/+/−	O	2 Y
Schellenberg 2018^[36]^	F	69	IDC	IIIC	+/+/−	O	C/R/E	Colon	2 Y	no	+/−/−	O	Die after 4 Y
You 2019^[37]^	F	43	DCIS	IA	+/+/+	O	E	Colon	2 Y	no	ND	E	23 M
De Gruttola 2019^[38]^	F	61	ILC	IIB	+/+/−	O	C/R/E	Stomach	8 Y	Gastric perforation	+/−/−	O	6 M
Guzmán-Calderón 2017^[39]^	F	65	ILC	ND	+/+/−	O	E	Rectum	3 Y	Rectal bleeding	+/+/−	O	ND
Gizzi 2015^[40]^	F	72	ILC	IIIA	+/+/−	O	C/R/E	Colon	11 Y	Abdomidal pain, diarrhea	+/+/−	O	2 Y
Wang 2018^[41]^	F	51	IDC	IIA	+/+/−	O	C/E	Duodenum	8 Y	NAUSEA, vomiting	+/+/−	O	ND
Mohy-Ud-Din 2019^[42]^	F	83	ILC	1A	+/+/−	O	E	Stomach	10 Y	Nausea, vomiting	+/+/−	ND	ND

**Table 2:** The clinicopathological features of gastrointestinal metastases of breast carcinoma.

NO. of cases	32		
Age	62.75±11.70 (39–84)		
Sex			
Male	2 (6.25%)		
Female	30 (93.75%)		
Type of breast cancer (28/32)		Metastasis location (32/32)	
ILC	18 (64.29%)	Esophagus	4 (12.5%)
IDC	9 (32.14%)	Stomach	12 (37.5%)
DCIS	1 (3.57%)	Duodenum	1 (3.13%)
Stage of breast cancer (20/32)		Small bowel	4 (12.5%)
Stage I	3 (15%)	Appendix	1 (3.13%)
Stage II	7 (35%)	Colon	9 (28.13%)
Stage III	7 (35%)	Rectum	3 (9.38%)
Stage IV	3 (15%)	Interval time of metastasis (Y) (31/32)	9.62 ± 6.92 (0.08–24)
Receptor of breast cancer (26/32)		Receptor of GI metastasis (30/32)	
ER	25 (96.15%)	ER	26 (86.67%)
PR	20 (76.92%)	PR	14 (46.67%)
HER2	3 (11.53%)	HER2	2 (6.67%)
Treatment of breast cancer (31/32)		Treatment of GI metastasis(30/32)	
Operation	30 (96.77%)	operation	19 (63.33%)
Conservative	1 (3.23%)	Chemotherapy (C)	6 (20%)
AT after operation (29/32)		Endocrine therapy (E)	3 (10%)
Chemotherapy (C)	18 (62.07%)	Radiation (R)	6 (20%)
Endocrine therapy (E)	22 (75.86%)	Time of follow-up (M) (19/32)	25.53 ± 20.32 (2–60)
Radiation (R)	12 (41.38%)		

The clinical presentation of gastric metastases mimics that of a primary gastric tumor, and the endoscopic findings are nonspecific and may be difficult to distinguish from inflammatory lesions. Furthermore, gastric metastases are located in the submucosa, so sometimes the metastases are too deep to be accurately identified from an endoscopic biopsy. Among the 12 patients with gastric metastasis analyzed in our literature review, the main clinical symptom were epigastric discomfort and pain, except 3 patients who were admitted with gastric perforation. All of the abovementioned factors lead to difficulties in distinguishing gastric metastases from primary gastric tumors. The clinical presentation and endoscopic findings in our case were also unspecific.

Because the therapeutic approach completely differs, it is important to distinguish between metastatic disease and primary gastric cancer. Endoscopic biopsy and histopathological examinations are particularly important for the diagnosis, while immunohistochemistry is considered to be the gold standard for differentiating between metastatic and primary cancers.^[43,44]^ ER and PR, 2 important indicators of endocrine therapy and prognostic indicators of breast cancer, are the most frequently examined markers in such cases. ER and PR positivity is suggestive of metastatic disease from breast cancer, but both ER and PR can be positive in 32% and 12% of patients with cancer of a gastric origin, respectively, which indicates these receptors cannot represent suitable biomarkers to discriminate between breast cancer metastasis to the stomach and primary gastric cancer.^[45]^ In our literature review, the positive rates of ER and PR in the metastatic location were as high as 86.67% and 46.67%, respectively. GCDFP15 has been proven an accurate biomarker for identifying a malignant lesion of breast origin, yielding 55% to 76% sensitivity, and 95% to 100% specificity.^[21]^ Additionally, it is notably that GATA3, a member of the GATA family of zinc-finger DNA binding proteins, which is currently considered a reliable, sensitive, and specific immunomarker for the diagnosis of breast cancer, as it was found only in breast and urothelial carcinomas but not in other tumors.^[46]^ Lastly, we combined IHC for CK7 and CK20 as a useful tool to identify a malignant lesion of breast origin, as breast carcinomas are CK7 positive in 90% of cases versus the 50% to 55% of primary gastric cancers, whereas CK20 is negative in all breast carcinomas and highly positive in gastric, colorectal, and pancreatic carcinomas.^[47]^ When breast cancer metastasis to the gastrointestinal tract is suspected, positive immunohistochemistry for CK7, GCDFP15, and GATA3 can effectively confirm the diagnosis, especially in case of CK20 negativity. ER, CK7, and CDFP15 positivity as well as PR, CK20, and mammaglobin negativity suggest that a gastric lesion has a breast origin in our patient.

The mechanism of gastric metastasis of breast cancer is still unclear. It is important to mention the new role of chemokines in the mechanisms of tumor progression and metastasis.^[48]^ Chemokines are a superfamily of chemotactic cytokines present in organs that act as specific modulators of leukocyte migration to sites of inflammation and are involved in the initiation and promotion of carcinogenesis by providing growth and angiogenic factors. Currently, more than 40 chemokines and 18 receptors have been identified.^[49]^ Chemokine receptors CXCR4 and CXCR7 are highly expressed in breast cancer cells and are responsible for chemotaxis to certain target organs, such as lymph nodes. Therefore, these chemokines are supposed to play an important role in the mobilization, trafficking, and homing of cancer stem cells into metastatic sites.^[26,50]^ However, the expression of chemokines is passive, so distant metastasis may be encoded by the gene sequence of primary cancer, and more studies are needed to further clarify the role of chemokines in the formation of gastric metastasis from breast cancer. Moreover, Gurzu et al^[51]^ reported that CD44/HER2 positivity in ductal carcinoma cells might indicate a high risk of distant metastasis and a low response to chemotherapy.

Because of the lack of randomized trials and the lack of reports, there is no consensus in the medical fraternity about the management of breast cancer metastasizing to the gastrointestinal tract. There are several treatment modalities available, including systemic chemotherapy, hormonal therapy, and surgical procedure. However, majority of the patients with gastric metastases from breast cancer usually receive chemotherapy or hormone therapy, based on the assessment of hormone receptor statuses in metastatic tissues.^[21]^ But, symptoms like intestinal obstruction, hemorrhage, or perforation necessitate surgical involvement. In our review of the literature, 19 patients with gastrointestinal metastasis of breast cancer received surgical treatment, mainly including obstruction (7 cases), perforation (3 cases), bleeding (4 cases), and dysphagia (1 case). McLemore et al reported in their study that the average survival of metastasis to the gastrointestinal tract from primary breast cancer was 28 months, with chemotherapy and tamoxifen treatment having a significant influence.^[52]^ The time of follow-up in our review was 25.53 months. Considering a widespread metastasis of stomach without obstruction or bleeding, a combination of radiotherapy and hormone therapy was used to treat our patient, and surgical treatment was not necessary. At present, the abdominal symptoms were partially relieved during the 1-year follow-up, and further follow-up is also needed.

## 4 Conclusion

In conclusion, the clinical presentation of gastric metastases from breast cancer is inexpressive, and the endoscopic features also lack specificity. Therefore, immunohistochemistry is crucial and is the gold standard for differentiating between metastatic and primary cancers. Moreover, chemokines play important roles in tumor progression and metastasis. The most important point is that our case report supports the fact that considering the possibility of gastric metastases from breast cancer and performing an upper endoscopy are crucial for patients who present with any subtle gastric symptoms and have a past medical history of breast cancer, especially invasive lobular carcinoma, even if the breast cancer occurred more than 10 years ago.

## Author contributions

**Data curation:** Ji-Xin Fu.

**Methodology:** Long Li.

**Project administration:** Ya-Nan Zou.

**Writing – original draft:** Ji-Xin Fu.

**Writing – review & editing:** Xin-Jian Wang.
